# Verifications

**Published:** 1887-11

**Authors:** Flora A. Waddell


### VERIFICATIONS.

(Read before the Ohio State Medical Society, Cleveland, Ohio, May 10th and 11th, 1887, by Flora A. Waddell, M. D.)

While attending a course of lectures at Hahnemann College, Chicago, a year ago last winter, I studied *Materia Medica* my spare hours in the office of Professor W. S. Gee. On going to the desk one morning I found the February number of THE HOMŒOPATHIC PHYSICIAN. Having never seen a copy of it before, I began to examine the book somewhat closely to see what it might contain that was good. On page 59 I came upon ten symptoms, all belonging to different remedies. The editor requested the readers to return an answer, allowing them to use any of the repertories, but not any *Materia Medica*. Any one answering correctly all of questions was to receive the journal free for one year. Not intending to enter the list, I set out to find them for my own benefit, never having noticed but one of them anywhere before. I succeeded in about a week in finding all but one in Hering's *Condensed*. So much was said in the following journals about the symptoms being silly, such as you would never hear of, etc., that after I returned home I kept a record of what symptoms belonging to the list came under my care during the last year. The result is a verification of seven out of the ten symptoms; immediate relief followed by cure in every case.

Symptom No. 1. "Metrorrhagia of large black lumps, worse from any motion, with violent pains in groins and fear of death, despair, bright red face, and fever." Had treated the case before for the same trouble, getting relief, but it was not permanent. Now gave Coffee$^{200x}$, one dose dry on the tongue, putting a small quantity in one-half a glass of water to be given as needed. Only one more dose was given in an hour after the first. Permanent relief followed.

No. 2, verified in two cases, "Drawing, tearing pain in periosteum, worse at night in wet, stormy weather and when at rest; better by motion. Rhododendron$^{3x}$ cured.

No. 4, applied to a case of colic. Had been suffering twenty-four hours; had the usual allopathic drugging, the most prominent symptoms being the one in question. "Sensation as if something sharp kept rubbing and cutting in the abdomen, worse on any movement of the body." Cocculus$^{200x}$ (one dose) was given, repeated in half an hour. In one hour the patient was sleeping soundly, and awoke free from pain. No more medicine was needed.

No. 5 symptom occurred in a case of rheumatism of two years' duration. The man came limping into the office, saying: "Doctor, can you do anything for my limb? It distresses me so when I sit down that I cannot rest in that position at all." I said to him: "What is the feeling in your limb that prevents your resting in that position more than any other?"

To my surprise (for I never expected to hear of this symptom of all others mentioned) he said: "Whenever I sit down my limb feels as if broken off right here" (pointing to about the middle of his thigh). He went on to state that he often got up and straightened himself to see if such was not the case. He had some pain in his arm, quite bad in his back, which he described as a sore pain. Not having any Illicium (or Star Anise) I gave him Sac lac., and told him to call on Saturday and report. I was determined to give the remedy a fair trial, and sent to Chicago for some. Gave the 30x dilution on disks, two be taken every night at bedtime for a week, then report. At the end of the week reported much better. At the end of four weeks was entirely well, and has remained so for six months. He is able to work as well as he ever could.

No. 7, lady afflicted with a complication of rectal and uterine troubles. Looked the case over, but could get no marked symptoms to prescribe from. I sat studying what I should give, when she said : " I do wish you would give me something to warm my back, it is so cold, and I cannot get it warm, and it has spells of itching so, it almost sets me crazy." If I had not seen this symptom so marked in the journal I would have passed it by or not have known what to do with it. Now I gave Muriate of Ammonia$^{200x}$, one dose a day for four days, then one a week for six weeks, when the patient reported well, and has remained so ever since.

No. 8 symptom, " Gurgling feeling in shoulder-joint or a feeling as of something alive, especially about midnight," belongs to Berberis, and has been verified in quite a number of cases, the feeling occurring in different parts of the body."

No. 9, the last symptom on the list, I have never met with but once, occurring in a young man sent to me by another physician for treatment. I will refer to nothing but this one symptom, as it was the leading, and I have already detained you too long. A constant, irresistible desire to walk in the open air ; it does not fatigue. Fluoric acid$^{12x}$ cured the case. The remedies he had before coming to me I do not know.

